# Meaning-Events: The Sensorimotor Foundation for Lifespan Development of Meaning

**DOI:** 10.3390/bs16050642

**Published:** 2026-04-24

**Authors:** Julia Penn Shaw

**Affiliations:** Department of Psychology and Human Development, College of Social and Behavioral Sciences, Empire State University, Saratoga Springs, NY 12866, USA; julie.shaw@sunyempire.edu

**Keywords:** meaning-events, joint attention, embodied cognition, dynamic skill theory, lifespan development, sensorimotor coordination, meaning-making, psychosocial development

## Abstract

Despite broad agreement on sensorimotor foundations of cognition, existing integrative models are not user-friendly to those who most need them—parents, caregivers, teachers, and clinical practitioners. This review addresses that gap by proposing Meaning-Events (M-Es) as sensorimotor–cognitive coordination units that structure meaning from infancy through adulthood. Drawing on joint attention research, embodied cognition, and dynamic skill theory, this integrative model demonstrates how sensorimotor processes—gaze coordination, affective timing/synchrony, bodily orientation, eye–hand coordination, and goal-directed action—organize cognitive structures of increasingly abstract meaning-making. Meaning-Events are shown as the smallest analyzable units that integrate sensorimotor experience with cognition, providing (1) developmental continuity for embodied action giving rise to coherent thought, purpose, and identity; (2) reciprocal perspectives informing impacts of dyadic behavioral interactions; and (3) an analytical and synthetic tool providing visible, measurable differentiation and integration of behaviors over time. Integration of Fischer’s dynamic skill theory with Erikson’s psychosocial theory illustrates applications in clinical and educational contexts. Rather than viewing sensorimotor experience as an early precursor superseded by symbolic cognition, the Meaning-Event model positions these sensorimotor–cognitive coordination units as constitutive of meaning at all developmental levels.

## 1. Background, Rationale, and Scope

Within the behavioral sciences, sensorimotor experiences are increasingly recognized as foundational to cognition, social understanding, and meaning-making (Siposova & Carpenter, 2019; Pexman, 2019; De Felice et al., 2025; de Barbaro et al., 2016). From infancy onward, human beings cognize the world through coordinated bodily actions, perceptual alignment, and affective regulation within social contexts (Gallagher, 2020; Piaget, 1952; Thelen & Smith, 1994; Barsalou, 2008, 2020). Research in embodied cognition, developmental systems theory, and social interaction demonstrates that perception, movement, and emotion actively organize cognition rather than being peripheral to it (Kontra et al., 2015; Lerner & Overton, 2017; Varela et al., 1991; Glenberg, 2015; Overton, 2015). Yet despite broad agreement on sensorimotor foundations of cognition, existing integrative models are not user-friendly to those who might most need them to analyze their interactions with others, such as parents, child and elder care workers, teachers, and clinical practitioners.

This paper addresses that gap by proposing Meaning-Events (M-Es) as sensorimotor–cognitive coordination units that structure meaning from infancy through adulthood. Meaning-Events are grounded in bodily processes expressed by two individuals in relationship: gaze following, postural alignment, rhythmic timing, emotional attunement, and goal-directed action. These sensorimotor dynamics—first evident in early interactions—are progressively differentiated and integrated, unique to each partner, into higher-order cognitive and psychosocial structures that undergird meaning-making.

Existing developmental frameworks have not captured, in a visible and easily understandable way, how sensorimotor processes and higher-order meaning connect, especially in interaction with another. Cognitive developmental theories emphasize increasing abstraction and representational complexity (Fischer, 1980; Mascolo, 2020a; Fischer & Bidell, 2006), while psychosocial theories focus on identity formation and meaning within social contexts (Erikson, 1950, 1963). What is missing is an easily understood and easily shared structural unit that accounts for how bodily action, emotion, cognition, and context are coordinated within related experiences, and reorganized across time. The Meaning-Event model—based on joint attention—fills this need.

Joint attention, as the foundation for Meaning-Events, provides a particularly well-established entry point for understanding developmental continuity as influenced by bounded experiences of sensory–cognitive coordination between two meaning-makers. The structure of the M-E could be extended to joint intention and joint action, as well, but those extensions will not be pursued in this paper. Classic and contemporary research demonstrates that shared attention emerges through tightly coordinated sensorimotor processes involving eye movements, manual actions, affective timing, and bodily orientation between interacting partners (Çetinçelik et al., 2020; Yu & Smith, 2017a; Scaife & Bruner, 1975; Tomasello & Farrar, 1986; Moore & Dunham, 2014). Advances in developmental and social neuroscience further indicate that these processes recruit distributed neural systems supporting perception–action coupling, affect regulation, and goal representation (Brandone, 2015; Jones & Carr, 2004; Schilbach et al., 2013). Importantly, disruptions to these sensorimotor coordination processes compromise not only early social interaction but also later language development, symbolic thought, and shared meaning (Baldassano et al., 2017; Shin et al., 2021; Bruinsma et al., 2004; Sodian & Kristen-Antonow, 2015).

Together, these findings suggest that sensorimotor–cognitive coordination in a Meaning-Event is a persistent organizing principle of cognition. This structure captures both micro-level sensorimotor dynamics—gaze coordination and affective synchrony—and macro-level developmental processes such as reflective meaning and identity formation.

This article develops the model with a focus on the sensorimotor foundations of developmental continuity. First, it clarifies how Meaning-Events transform ongoing sensorimotor engagement into discrete, analyzable units. Second, it demonstrates how the framework aligns with dynamic skill theory to explain differentiation and integration across development (Fischer, 1980; Fischer & Bidell, 2006; Mascolo, 2020a). Third, it shows how Meaning-Events support psychosocial integration without abandoning embodied grounding (Erikson, 1950, 1963). Finally, it explores implications for research, clinical practice, and education. By framing meaning as a consistent embodied, sensorimotor-coordinated process, the model contributes to efforts unifying sensorimotor, cognitive, and developmental perspectives as meaning becomes increasingly abstract and socially extended.

## 2. Theoretical Foundations for Meaning-Events: From Amorphous Sensory Experiences to Structured Units of Meaning

Meaning is among the most widely used yet theoretically diffuse constructs in the behavioral sciences. Contemporary approaches identify meaning in life along dimensions of coherence, purpose, and significance (Martela & Steger, 2016) and mattering (Costin & Vignoles, 2020; George & Park, 2016, 2017). Although frequently treated as reflective or narrative achievements, developmental research suggests that meaningful events emerge through ongoing engagement with the world, organized by perception, action, emotion, and social interaction in conjunction with abstract representation (Feldman, 2017; Piaget, 1952; Varela et al., 1991; Thelen & Smith, 1994). From a developmental perspective, meaning is constructed in activity itself, as sensorimotor engagement becomes organized into increasingly stable and shareable forms (Barsalou, 2008, 2020; Overton, 2015). The Meaning-Event framework proposes that meaning arises through shared bounded experiences of sensorimotor–cognitive coordination that transform continuous experience into discrete units—called events—that can be remembered, communicated, and integrated over time.

Events are already understood by the general public and therefore much easier to apply to everyday life than are principles of mathematical group theory (Piaget, 1952) or set theory (Fischer, 1980). However, because the Meaning-Event structure is a subset of Fischer’s theory, it has the strength of set theory while being more understandable (Shaw, 2002, 2022).

### 2.1. Events as the Cognitive Organization of Sensorimotor Experience

Human experience unfolds continuously, yet it is not experienced as an undifferentiated stream. Research demonstrates that perception and action are organized into events—bounded units marked by changes in movement, attention, affect, or goal structure (Zacks, 2020; Richmond & Zacks, 2017; Tranel & Damasio, 2002; Zacks & Swallow, 2007; Radvansky & Zacks, 2017). Event theory proposes that individuals parse ongoing activity into discrete experiences to regulate prediction, memory, and action. This segmentation reflects underlying sensorimotor and predictive processes, not just symbolic categorization. Meaning does not emerge by adding interpretation to events but rather emerges when sensorimotor engagement becomes bounded and socially coordinated, allowing experience to be shared, regulated, and retained (Hipólito & van Es, 2022; Baldassano et al., 2017, 2018; Tomasello et al., 2005; Overton, 2015).

A key mechanism underlying this event parsing is statistical learning—the ability to detect regularities in continuous sensory input across time (C. Monroy et al., 2017; C. D. Monroy et al., 2018). Research demonstrates that infants and toddlers are sensitive to the statistical regularities embedded within action sequences. When certain actions consistently follow one another, observers learn the transitional probabilities between action steps and, as a result, generate anticipatory gaze toward upcoming actions before they occur (C. D. Monroy et al., 2017, 2019a). This capacity for statistical learning operates not merely as abstract pattern detection but as an action-oriented process: infants learn regularities more robustly when sequences are performed by a human agent than when they are presented as self-propelled non-social events. This suggests that the social and goal-directed structure of action sequences is integral to how statistical regularities are encoded (C. D. Monroy et al., 2017; C. Monroy et al., 2017). From a Meaning-Event perspective, statistical learning is evidence of the cognitive processes that segment continuous sensorimotor experience into bounded, anticipatable units—the very process that allows primitive sensorimotor engagement to crystallize into a Meaning-Event.

**Figure 1:**
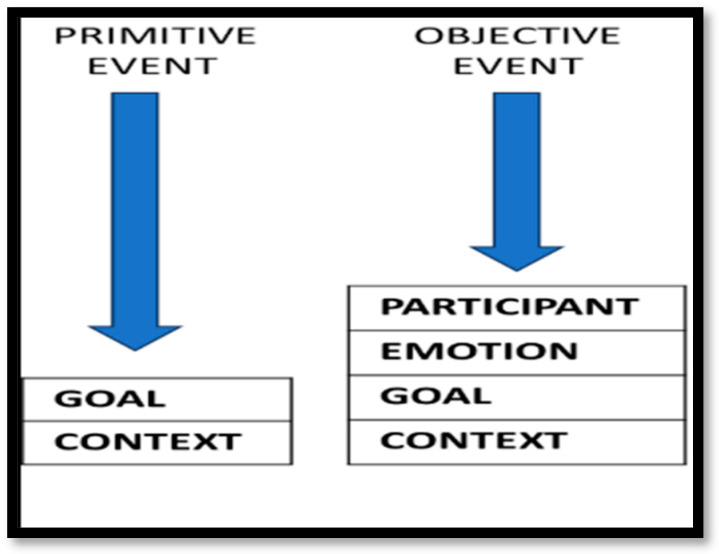
Comparing complexity of Primitive and Objective Event structures.

Three event descriptors are coined for this paper, varying in complexity and informational richness: Primitive Events, Objective Events, and Meaning-Events. First, *Primitive Events* encode what occurs and where or when it occurs, such as in an observed action or calendar-scheduled activity. Second, *Objective Events* incorporate information about participants and outcomes, as in newspaper reporting or observational description. See Figure 1 for a comparison of Primitive and Objective Event structures.

### 2.2. Joint Attention as a Sensorimotor Mechanism for Meaning-Events: An Illustrative Example

The third event descriptor is for *Meaning-Events*. The premise of this paper is that typical depictions of events, portrayed as Primitive Events and Objective Events, do not capture the essence of events—and that if we have a fuller view of an event in mind, based on the importance of joint attention in significant interactions, then communication about many aspects of events will become easier. This matters because much human communication centers around descriptions of, interpretations of, and uses of events.

**Figure 2:**
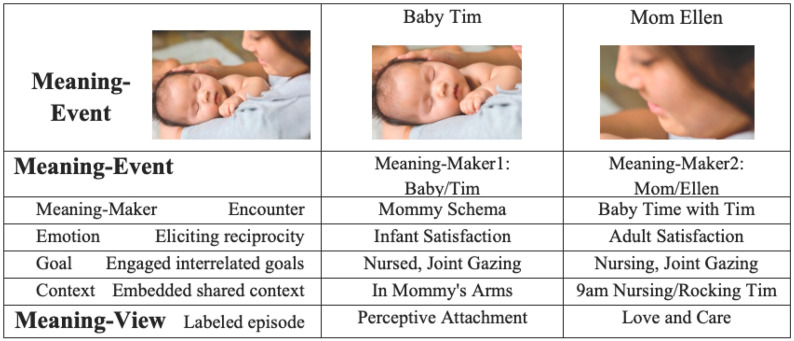
An illustrative Meaning-Event: an early caregiving experience.

The caregiving interaction in Figure 2 exemplifies that the biological foundation of Meaning-Events lies in joint attention—the shared focus of two individuals on a common object, action, or event—long recognized as a cornerstone of social cognition and language development (Kahl & Kopp, 2018; Wass et al., 2018b; De Felice et al., 2025; Yu & Smith, 2017a; Gredebäck & Falck-Ytter, 2015; Scaife & Bruner, 1975; Tomasello & Farrar, 1986; Butterworth, 2004). Joint attention is not merely shared reference; it is a sensorimotor achievement involving coordinated gaze, bodily orientation, manual action, temporal timing, and affective regulation (Carozza & Leong, 2021; Corbetta & Fagard, 2017; Çetinçelik et al., 2020; Davis et al., 2018; Meyer, 2018; Barrett, 2017; Suarez-Rivera et al., 2019; Adolph & Hoch, 2019; Moore & Dunham, 2014). Coordinated attention is achieved through multiple sensorimotor pathways that change developmentally (Yu & Smith, 2017a), recruiting distributed neural systems supporting perception–action coupling, affective resonance, and goal representation (Jones & Carr, 2004; Schilbach et al., 2013).

Each Meaning-Event comprises four analytically distinguishable but dynamically interdependent elements: two meaning-makers and the emotions, goals, and contexts for each. These elements are not independent components assembled after the fact; they are individually experienced through sensorimotor activity in interaction with another.

Meaning-makers are participants in an encounter whose perceptual orientations, bodily actions, and affective states shape the unfolding event. Meaning does not reside solely in individual perspectives but emerges through the coordination of at least two meaning-makers, whose actions and responses mutually constrain and organize the interaction (Tomasello & Farrar, 1986; Moore & Dunham, 2014).

Emotions function as regulatory processes within the Meaning-Event as the meaning-makers elicit emotions from each other. Emotional states shape attention, posture, timing, and readiness for action, signaling the relevance or significance of what is occurring (Çetinçelik et al., 2020; Sroufe, 2005; Schore, 2001). Emotion is therefore not an add-on to meaning but a core organizing force that modulates sensorimotor coordination within the event. Viewing the emotional responses of each of the two meaning-makers within the context of each other in the M-E structure enables an analysis of both interoception (Schaan et al., 2019) and intersubjectivity (Mascolo, 2020b).

Goals reflect the directed nature of action within a Meaning-Event. The meaning-makers engage in interrelated goals, which may be explicit or implicit, articulated or embodied in patterns of movement and attention. Goal orientation informs how activity is organized over time, guiding the coordination of perception and action toward anticipated outcomes (Mascolo & Fischer, 2015).

Contexts situate the Meaning-Event within spatial, temporal, social, and cultural constraints. The embedded shared—and also personal—contexts brought by each meaning-maker to the M-E shape what actions are possible, how they are interpreted, and how sensorimotor cues are understood. Importantly, context is not merely external; it is enacted through the meaning-makers’ orientation to place, time, roles, and expectations (Bronfenbrenner, 1979; Overton, 2015).

Meaning arises through the coordination of these four elements. It is this coordination—enacted through sensorimotor processes—that transforms activity into an event experienced as eventful. By structuring all of the elements of the event into one unit, the Meaning-Event framework models the integration of biological, cognitive, and psychosocial perspectives of a particular meaning-maker in relation to another particular meaning-maker. Meaning-Events are empirically accessible through observable sensorimotor variables—gaze coordination, posture, affective timing, and goal-directed action—as well as gestures, words, and symbols that describe both external aspects and subjective interpretation of the event. The interpretation of this coordination is expressed as the Meaning-View—unique to each participating meaning-maker, and also to each observer. This important aspect of the Meaning-Event is captured in the Meaning-View, discussed in Section 3.

### 2.3. The Meaning-Event Model

**Figure 3:**
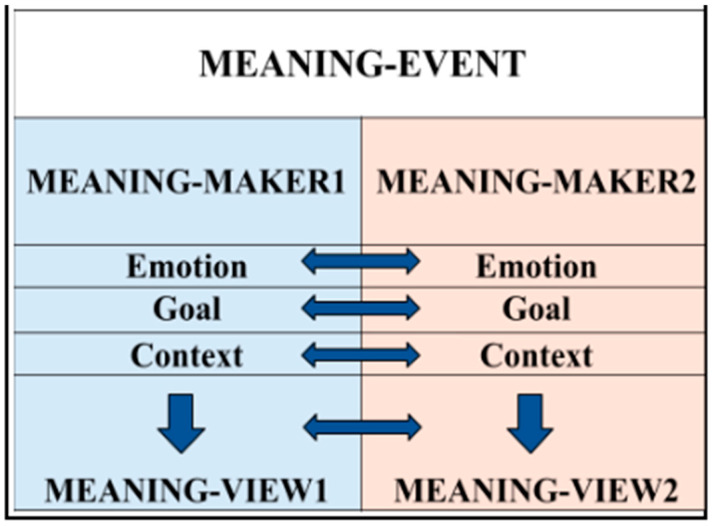
The Meaning-Event model as an abstraction.

Meaning-Events are bounded experiences of sensorimotor–cognitive coordination between two meaning-makers, in which attention, action, affect, and intention are aligned within a shared context. They are enacted through bodily orientation, gaze, timing, emotional regulation, and goal-directed activity (Feldman, 2017; Yu & Smith, 2017a; Scaife & Bruner, 1975; Tomasello et al., 2005; Moore & Dunham, 2014). Meaning-Events thus provide a structural account of how sensorimotor processes are organized into coherent experiences that can be remembered, communicated, and integrated as meaningful. The Meaning-Event model, as shown in Figure 3, specifies how sensorimotor coordination becomes integrated into stable units of meaning across development. The model treats meaning as an organized outcome of embodied interaction (Newen et al., 2018). The model represents two Meaning-Makers interacting—each in a different color. The arrows between the Emotions, the Goals, and the Contexts of each Meaning-Maker highlight their interactions as they relate to each other. The wider downward arrows between these relationships and the Meaning-Views indicates that a holistic meta-level Meaning-View emerges for each Meaning-Maker as a result of the interactions – and that these Meaning-Views also relate to each other. 

The Meaning-Event structure is a transformation of the event structure itself. Meaning-Events extend the usefulness of event structure well beyond the Primitive Event or the Objective Event by incorporating the subjective and interpersonal experiences of emotion, intention, and contextual interpretation between interacting individuals (Fast et al., 2013; Radvansky & Zacks, 2017).

Critically, the Meaning-Event structure is not merely a snapshot, but a temporally unfolding sequence of coordinated actions. Research on action-sequence processing demonstrates that both infants and adults parse continuous streams of behavior into structured units whose internal ordering carries predictive weight (C. D. Monroy et al., 2017, 2019b). EEG studies reveal that violations to learned action-pair regularities elicit distinct neural responses—including a negative central component indexing visual attention—when the structural integrity of the action sequence is broken (C. D. Monroy et al., 2019b). These findings suggest that the sequential structure within a Meaning-Event is not incidental but constitutive: the meaning of any action step is shaped by what preceded it and what is anticipated to follow. Moreover, the infant motor system actively uses knowledge acquired through observational statistical learning to generate predictive motor activation prior to the next action in a sequence (C. D. Monroy et al., 2019a), indicating that Meaning-Event formation engages not only perceptual but also motoric anticipation. Chen and Monroy (2026) extend this perspective across sensory modalities, reviewing how motor, visual, auditory, olfactory, and gustatory experiences collectively shape early cognitive development through predictive processing—providing broad convergent support for the sensorimotor foundations of Meaning-Event formation.

The Meaning-Event demonstrates how sensorimotor experience becomes organized into discrete, developmentally consequential units, providing a systematic framework for understanding how early sensory and motor engagement supports learning, language, memory, and identity in both typical and atypical development. Meaning-Events can visualize specific disruptions to joint attention that compromise not only early social interaction but later language, symbolic thought, and shared meaning, as evidenced in autism spectrum conditions (Bruinsma et al., 2004; Sodian & Kristen-Antonow, 2015). Robbie Case provided evidence that development occurs through the increasing capacity of the child’s working memory and through the increase in operational efficiency of memory (Case, 1987, 2013).

## 3. Integrative Meaning-Views Emerge from Each Meaning-Event

A Meaning-View is a stable gestalt of the ‘meaning’ of a Meaning-Event created by a meaning-maker (whether a participant in the M-E or an observer), when all four of the Meaning-Event elements—the meaning-makers, emotions, goals, and contexts—are independently stored and available for intercoordination with all other elements into what Fischer (1980) calls a developmental tier (a system of systems of skills). If one of the elements is changed in a subsequent Meaning-Event, such as a new goal or new context, the impact of that change may lead to a modified Meaning-View.

Each Meaning-View persists beyond the immediate interaction and shapes subsequent perception and action (Tranel & Damasio, 2002; Fonagy & Target, 1996). A Meaning-View becomes part of the individual’s available repertoire of elements for future Meaning-Events. It shapes expectations, attention, emotional readiness, and goal orientation in subsequent interactions, enabling—but also constraining—complex forms of coordination (Fonagy et al., 2002).

Because Meaning-Views retain their sensorimotor grounding, even abstract or reflective meaning remains linked to bodily processes such as timing, gesture, affective attunement, and spatial orientation (Seth & Friston, 2016; Glenberg, 2015). For example, when a Meaning-View is one of secure attachment, an individual is likely to trust future interactions, and this trust will be evident in sensorimotor responses such as shared gazing and smiling.

Importantly, Meaning-Views at all developmental levels contribute to later expectations regarding social engagement, emotional regulation, and interpersonal meaning, illustrating how early sensorimotor experience continues to shape cognition and meaning across development (Redcay & Schilbach, 2019; Erikson, 1950, 1963; Fonagy et al., 2002). Because M-Es—with their associated Meaning-Views—are visual structures, they can help to analyze and predict developmental progression, without developmental determinism (Ben-Yakov & Henson, 2018; Ezzyat & Davachi, 2021; Friston et al., 2017; Gilboa & Marlatte, 2017; DuBrow & Davachi, 2016; Clewett & Davachi, 2017; Loeffler et al., 2016). Adult conversation, therapeutic interaction, and educational learning all depend on Meaning-Views arising from specific prior Meaning-Event experiences, often operating below conscious awareness.

Meaning-Views are recursive developmental structures. Each new Meaning-Event both draws upon and reshapes existing Meaning-Views, allowing development through iterative cycles of integration and reorganization (Kahl & Kopp, 2018; McAdams, 2018; Crocetti, 2017; Schlichting & Preston, 2015; Granott & Parziale, 2002; Mascolo, 2020a). From a dynamic skill perspective, a Meaning-View results when the four elements of the Meaning-Event become coordinated as a system of systems of skills at a prior developmental tier, and this Meaning-View influences the elements for higher-order Meaning-Event systems (Behrens et al., 2018; Hasson et al., 2015; Fischer, 1980; Fischer & Bidell, 2006; Mascolo, 2020a; Granott & Parziale, 2002) and for broader relational systems as well (Paley & Hajal, 2022).

This recursive process supports increasing complexity. Early Meaning-Views grounded in bodily regulation and shared attention integrate into later Meaning-Views involving symbolic reasoning, moral judgment, and identity construction. At each stage, sensorimotor–cognitive coordination patterns might be preserved or reorganized, accommodating both developmental continuity and transformation.

## 4. Lifespan Development of Meaning: Differentiation and Integration of Sensorimotor–Cognitive Coordination Within Meaning-Event Units

Following Werner and Kaplan (1984), development proceeds from global, undifferentiated arrangements toward increasingly differentiated and hierarchically integrated systems. This principle does not describe movement away from sensorimotor experience, but rather its recursive reorganization into progressively more complex forms of cognitive and psychosocial meaning.

The Meaning-Event structure enhances differentiation of development and integration of development across the lifespan by linking two complementary lifespan developmental frameworks: first, Fischer’s dynamic skill theory, which articulates both differentiation and integration of cognitive complexity, and second, Erikson’s psychosocial theory, which addresses the integration of meaning within identity and social life. Meaning-Events function as the common mechanism through which sensorimotor coordination is organized and reorganized across both dimensions. Meaning-Events are dominated by overt sensorimotor coordination: gaze alignment, bodily orientation, affective regulation, and goal-directed action. As development proceeds, these coordination processes become increasingly internalized, distributed, and symbolically scaffolded, expanding in complexity while remaining grounded in embodied engagement (Fischer, 1980; Piaget, 1952; Overton, 2015; Glenberg, 2015).

### 4.1. Differentiation and Kurt Fischer’s Dynamic Skill Theory

Fischer’s dynamic skill theory uses mathematical set theory to demonstrate how skills (sets) become more complex with development. Fischer defines four lifespan developmental tiers whose names indicate the complexity of skills being developed—Sensorimotor Action (~3 months–2 years), Representation (~age 2 to age 10), Abstract (~age 10–age 20), and Principle (~age 20 and older). Higher developmental tiers build upon skills learned at lower developmental tiers.

The Meaning-Event model arose from and aligns well with Kurt Fischer (Shaw, 2002, 2022). The specific skills addressed here are the elements related to joint attention (element = skill): meaning-makers, emotions, goals, and contexts. Elements are identified by their tier: e.g., action-element; representation-element; abstract-element; or principle-element.

**Fischer Developmental Tiers:**

- Action Tier: Sensory and motor actions, such as gazing, reaching, and smiling, are action-elements that coordinate to achieve joint attention.
- Representation Tier: Words/symbols such as ‘mom’, ‘hug’, ‘bottle’, and ‘kitchen’ are representation-elements that coordinate sensorimotor actions learned at the Action Tier.
- Abstract Tier: Concepts such as ‘family’, ‘sharing’, ‘event’, and ‘home’ are abstract-elements that coordinate representations learned at the Representation Tier.
- Principle Tier: Principles such as ‘universal love’, ‘joint attention’, or ‘developmental theory’ are principle-elements that coordinate concepts learned at the Abstract Tier.

Coordination of joint attention elements occurs as four levels of transformation within each tier. This example is coordination of elements from the Action Tier to the Representational Tier:

- Single Element: Individual elements differentiate within a developmental tier. *Action Tier example:* Baby coordinates sensorimotor actions with its mother, as ‘mom’.
- Mapped Elements: Two single action-elements coordinate. *Example:* When ‘Mom’ and ‘eating’ are mapped, the child responds to various scenarios of eating with mom.
- System of Elements: Two mappings of elements coordinate. *Example:* Eating with mom, and eating with others, coordinate to scenarios of eating with different people having different emotions in different contexts.
- System of System of Elements at Tier^N^ = Single Element at Tier^N+1^: All four elements of joint attention are coordinated. *Example:* The child engages with different people with different emotions in different settings for different goals, learning words to associate with the four elements of these many joint attention events. Each word learned is a Meaning-View, or a system of systems of elements on the Action Tier and a single element at the subsequent Representation Tier.

Coordinating the four elements within a tier is very complex: Four specific single elements (4) + the number of mappings (6) + the number of systems of those elements (3) + system of systems of those elements (1) = 14 discrete coordination patterns. This illustrates the considerable number of specific interactions required by a child to move from one developmental tier to another.

Dynamic skill theory provides a detailed account of how cognitive structures emerge through the coordination of simpler skills into increasingly complex systems across a tier. These tiers allow earlier skills to function within more complex systems, allowing coordination of attention, intention, and affect without immediate action (Fischer, 1980; Fischer & Bidell, 2006; Mascolo, 2020a; Spencer et al., 2011; Perone & Simmering, 2017; Granott & Parziale, 2002; Barsalou, 2008, 2020; Glenberg, 2015).

### 4.2. Integration and Erikson’s Psychosocial Development

While dynamic skill theory was used here to account for differentiation, it can also enhance Erikson’s theory that addresses the integration of meaning within personal identity formation in social/cultural contexts (Erikson, 1950, 1963). Erikson conceptualized development as a sequence of psychosocial challenges, each involving integration of personal capacities within social expectations. He presents each challenge as a dichotomy—very different results follow if the challenge is met or not—starting with trust vs. mistrust in infancy. The Meaning-Event model, as integrated with Erikson’s theory in Table 1 below, suggests that an Erikson stage is resolved when milestone Meaning-Events lead to a transition in a person’s global Meaning-View, which we recall is an integration of the elements within a Meaning-Event at the next highest developmental tier. Erikson’s theory captures the ‘meaning’ of Fischer’s developmental process. This is a complex topic and cannot thoroughly be addressed here. 

Ideally, each psychosocial stage reflects success with age-related embodied positive coordinations of Meaning-Event elements. For example, early trust and mistrust emerge through sensorimotor regulation of comfort, arousal, and affect within caregiving interactions (Higgins et al., 2021; Sroufe, 2005; Tronick, 2017). Subsequent stages involving autonomy, initiative, and industry are negotiated through coordinated interaction with objects, tools, and people, requiring increasingly refined control of movement, timing, and attention (De Felice et al., 2025). In adolescence and adulthood, identity formation, intimacy, generativity, and integrity involve the integration of prior Meaning-Views into broader narratives of self, purpose, and belonging (Dunlop, 2021; Costello & Bloesch, 2017; De Moor, 2023; Syed & McLean, 2016; Branje, 2022; Branje et al., 2021; McAdams, 2018, 2019; McAdams & McLean, 2013).

These later forms of meaning have both continuities based on stability of prior Meaning-Events, and the capacity to change in conjunction with new sensorimotor–cognitive perspectives of the meaning-makers, emotions, goals, and contexts within new Meaning-Events. Meaning-Events thus provide the mechanism through which psychosocial status can be analyzed, sometimes to preserve continuity between early embodied experience and later reflective meaning, and sometimes to enable change to Meaning-Views of past Meaning-Events.

By foregrounding sensorimotor–cognitive coordination as a consistent developmental mechanism, the framework aligns with efforts to understand how early sensory and motor engagement shapes lifelong learning, attention, memory, language, and identity (Atzil et al., 2018; Kuehn et al., 2018; Loeffler et al., 2016; Planalp & Braungart-Rieker, 2015; Overton, 2015; Glenberg, 2015; Bandler & Grinder, 1975).

## 5. Implications and Applications of the Meaning-Event Framework

The Meaning-Event framework has implications for contexts where learning, development, and psychological well-being are central. Because Meaning-Events are units of sensorimotor–cognitive coordination, the framework offers a way to examine how bodily action, perceptual alignment, affective regulation, and goal-directed engagement organize meaning across development. The following applications briefly illustrate how Meaning-Events function as analytic lenses across clinical, educational, and developmental contexts.

### 5.1. Clinical Contexts: Meaning-Events Integrate Sensory Experiences Within Therapeutic Interaction

In clinical contexts, therapeutic change is increasingly understood as unfolding through interactions rather than insights alone. Research on psychotherapy emphasizes affect regulation, interpersonal timing, and embodied attunement in facilitating psychological integration (Booker et al., 2022; Luyten et al., 2020; Price & Hooven, 2018; Schilbach, 2016; Koole & Tschacher, 2016; Butler, 2015; Schilbach et al., 2013; Fonagy & Target, 1996; Fonagy et al., 2002; Allen, 2013). Therapeutic encounters can be understood as structured experiences reorganizing sensorimotor coordination, emotional regulation, and shared intentions within an M-E, potentially leading to healthier Meaning-Views.

Therapeutic Meaning-Events between client and clinician involve coordinated attention, posture, gesture, vocal tone, and affective timing, often operating below explicit verbal content (Wampold, 2015; Hu et al., 2017; Schore, 2001; Tronick, 2017). Difficulties in meaning-making may therefore reflect disruptions in the organization of Meaning-Events—such as mis-attunement, dysregulated affect, or rigid goal orientation—rather than deficits in symbolic understanding alone. Therapeutic change can be understood as supporting new Meaning-Views through reorganization of sensorimotor coordination, allowing previously destabilizing experiences to be integrated into more coherent forms of meaning (Fonagy et al., 2002).

**Example: Adolescent Misinterpretation of Intensity for Anger.** Joan, 38, is therapist for Alexander, 17, to help him address anger issues. In a session, Joan raises her voice to make a point; Alexander becomes upset because he thinks she is angry. Joan uses the Meaning-Event model to visualize this experience, showing that she got louder to focus on a statement. Visualizing the episode as a Meaning-Event—and thereby distancing it—helped Alexander to see that a louder tone does not always mean an angry intent (Monk et al., 2003).

### 5.2. Educational Contexts: Learning as Sensorimotor–Cognitive Coordination

Educational settings provide a clear illustration of how Meaning-Events support cognitive development through embodied engagement. Learning does not occur through symbolic instruction alone; it unfolds through coordinated activity involving attention, action, emotion, and shared focus between learners and educators in conjunction with skills in manipulating both objects and their symbolic representations (Castro-Alonso et al., 2024; Kontra et al., 2015; De Felice et al., 2025; Abrahamson et al., 2020; Piaget, 1952; Vygotsky, 1934/2012; Shaw & Chen, 2012; Tomasello & Farrar, 1986). Classroom interactions, demonstrations, collaborative problem-solving, clear labeling of interacting concepts, and guided practice can all be interpreted as making use of the Meaning-Event structure in learning.

In early education, this coordination is overt: through shared manipulation of objects, gesture-supported explanations, and embodied exploration (Nathan & Walkington, 2017; Montessori, 1912, Thelen & Smith, 1994). As learning becomes more abstract, sensorimotor processes remain active but increasingly implicit. Gesture supports reasoning; spatial orientation shapes problem-solving; temporal coordination structures turn-taking (Novack & Goldin-Meadow, 2015; Macedonia, 2014, 2019; Barsalou, 2020; Johnson-Glenberg, 2018; Barsalou, 2008; Glenberg, 2015). Meaning-Views formed through these interactions provide the basis for increasingly complex understanding, demonstrating how sensorimotor coordination remains a mechanism of learning even in symbolic domains such as mathematics and language. The M-E model highlights structured interactional engagement, pacing, and embodied engagement in educational design (Crocetti, 2017; Shaw & Chen, 2012; Syed & McLean, 2016).

**Example: Montessori Method of Instruction.** The child meaning-maker selects the task to which the teacher–guide attends, which is to organize blocks. The teacher–guide demonstrates placing the blocks in order of height and says ‘height’. The attentive teacher–guide meaning-maker, the reciprocal emotions, the child-selected task-level, and the child-sized context all speak to an effective Meaning-Event (Montessori, 1912).

### 5.3. Implications for Sensorily Grounded Developmental Research

Across clinical, educational, and research contexts, the Meaning-Event model emphasizes a common principle: sensorimotor coordination is a consistent mechanism of meaning-making, not a developmental stage that is superseded by symbolic cognition. Whether in early caregiving interactions, classroom learning, or adult therapeutic encounters, Meaning-Events organize experience by coordinating perception, action, affect, and intention within shared contexts that can be visualized, manipulated, and shared (Carollo et al., 2021; Morris et al., 2017; Schlichting & Preston, 2015).

By foregrounding this developmental continuity, the Meaning-Event model contributes to ongoing efforts to understand how early sensorimotor experience shapes cognition in both typical and atypical development. It offers an integrative framework that preserves the centrality of meaning while making its embodied foundations analytically visible.

The framework has implications for developmental research more broadly. By treating Meaning-Events as units organizing sensorimotor experience into cognitive and psychosocial meaning, the model connects micro-level interactional processes with macro-level developmental outcomes (Jones & Carr, 2004; Werner & Kaplan, 1984; Granott & Parziale, 2002). This is particularly relevant given methodological advances including dual head-mounted eye-tracking, fine-grained motion analysis, and multimodal behavioral coding, which make it possible to study how gaze coordination, bodily alignment, affective timing, and goal-directed action contribute to Meaning-Event formation (Higgins et al., 2021; Kahl & Kopp, 2018; Yu & Smith, 2017a; Overton, 2015).

Empirical research on the joint action of parent–infant dyads provides some of the most compelling evidence that the Meaning-Event structure maps onto observable behavior. Using head-mounted eye-tracking during live, free-flowing parent–infant play, C. Monroy et al. (2021a) demonstrated that 9-month-old infants anticipate their parents’ actions at rates significantly above chance, and that the frequency of these anticipations is correlated with child-led joint attention and hand–eye coordination. This finding is particularly relevant to the Meaning-Event framework: within the dyad, each meaning-maker’s sensorimotor orientation is continuously shaped by the other’s goals and actions, making action prediction a real-time expression of coordinated meaning-making. Further evidence comes from Chen et al. (2021), who showed that parent–toddler dyads in novel object play spontaneously modify their joint behavioral patterns—increasing the synchrony between parents’ naming and children’s object attention—precisely when novel referent mapping is required. These dyadic adjustments are not scripted but emerge from the mutual responsiveness of two meaning-makers whose goals, emotions, and attentional orientations are dynamically aligned within the Meaning-Event.

Relatedly, C. Monroy et al. (2024) found that parent–child sensorimotor coordination during joint tasks involves hand–eye coordination from both dyad members, with each partner’s motor behavior structuring the other’s visual attention—precisely the kind of bidirectional sensorimotor coupling the Meaning-Event model predicts. Chen et al. (2020) further showed that coordinated attention in parent–toddler interaction depends on multimodal behavioral contingencies that vary with the toddler’s hearing status, underscoring how sensorimotor differences between dyad members shape Meaning-Event quality. Slone et al. (2018) demonstrated with head-mounted eye-tracking that children’s dynamic visual attention during naturalistic behavior shifts rapidly and is tightly coupled with their own manual actions, providing further evidence for the sensorimotor integration that underpins Meaning-Event formation.

Because Meaning-Events are recursively integrated across development, the framework accommodates both stability and change. Early Meaning-Views that color initial sensorimotor experiences remain influential without determining developmental outcomes, allowing for reorganization and growth across the lifespan. Rather than static representations, Meaning-Events remain active organizers of perception, action, and interpretation (Tranel & Damasio, 2002; Fonagy & Target, 1996), enabling cycles of integration and reorganization (Lehmann et al., 2024; Booker et al., 2022; Hartog et al., 2020; Granott & Parziale, 2002).

## 6. Discussion: Sensorimotor Foundations of Meaning Across Development

This article addresses how early sensorimotor experience gives rise to increasingly complex forms of cognition and meaning across the lifespan. The Meaning-Event framework positions meaning as an organized outcome of sensorimotor coordination enacted within social interaction. The central claim is that meaning remains mechanistically grounded in sensorimotor processes throughout development. Meaning-Event units organize bodily action, perceptual alignment, affective regulation, and goal-directed engagement into coherent experiences supporting lifelong learning, memory, language, and identity (Kuehn et al., 2018; Pexman, 2019; Gallagher, 2020; De Felice et al., 2025; Barsalou, 2008, 2020; Glenberg, 2015; Overton, 2015). Symbolic and narrative meaning do not replace sensorimotor experience; they reorganize and extend it.

### 6.1. Sensorimotor Experience as a Consistent Developmental Mechanism

A recurring challenge in developmental theory is accounting for continuity between early embodied experience and later abstract cognition. Sensorimotor processes are acknowledged as foundational in infancy but often recede from view as representational capacities become the focus (Piaget, 1952; Vygotsky, 1934/2012). The Meaning-Event framework addresses this challenge by treating sensorimotor coordination as a persistent developmental mechanism, rather than a stage that is superseded.

Beyond its theoretical contributions, the Meaning-Event structure functions as an analytical tool for studying behavior. Because the four elements of the Meaning-Event—two meaning-makers, each with their own emotions, goals, and contexts—are operationally anchored in observable sensorimotor variables, they can be coded from naturalistic behavioral data. Research programs using head-mounted eye-tracking to capture first-person gaze during parent–infant interaction (C. Monroy et al., 2021a, 2024; Slone et al., 2018) demonstrate that micro-level behavioral variables—anticipatory fixations, hand–eye coordination bouts, joint attention episodes—can be reliably coded and analyzed as the building blocks of dyadic coordination. These are precisely the variables that constitute the sensorimotor substrate of a Meaning-Event. Applying the Meaning-Event framework to such datasets would allow researchers to ask not only ‘how much joint attention occurred’ but ‘how did the four elements of the Meaning-Event coordinate across the interaction, and what Meaning-View was likely generated?’ The framework also scales to statistical learning paradigms: the detection of regularities across action-sequence trials (C. D. Monroy et al., 2017, 2018, 2019b) can be re-analyzed as evidence for the formation and revision of Meaning-Views—stable anticipatory structures that persist beyond the immediate interaction and shape subsequent perception. Chen et al. (2018), studying cross-situational word learning across hierarchical levels, similarly showed that learners aggregate statistical regularities across distributional input to build object-name mappings, a process directly analogous to how Meaning-Views are constructed and refined across successive Meaning-Events. Taken together, these methodological convergences suggest that the Meaning-Event model is not only a conceptual framework but an empirically tractable unit of analysis, amenable to operationalization through existing behavioral and neurophysiological tools.

Joint attention structures this continuity. While its overt behavioral form changes with development, the underlying coordination processes remain active in later interaction, shaping turn-taking, gesture, emotional attunement, and shared intentionality in adulthood (Siposova & Carpenter, 2019; Redcay & Schilbach, 2019; Moore & Dunham, 2014; Yu & Smith, 2017a, 2017b). Meaning-Events preserve this coordination by organizing it into experiences increasingly mediated by language, symbols, and reflective thought.

### 6.2. Implications for Typical and Atypical Development

The framework has implications for both typical and atypical development. Because Meaning-Events can be identified through observable sensorimotor variables—gaze coordination, gesture, posture, affective timing—they offer an empirically tractable unit for studying how interaction shapes cognitive development (Whitall & Clark, 2018; Lord et al., 2018; Calkins & Perry, 2016; Tronick, 2017; Overton, 2015). This is particularly relevant in light of methodological advances, including dual head-mounted eye-tracking, fine-grained behavioral coding, and multimodal analysis (Yu & Smith, 2017a). M-Es can work in alignment with approaches such as Mindful Awareness in Body-oriented Therapy—MABT (Price & Hooven, 2018)—or be of use when analyzing parent–child dynamics (Ratliff et al., 2022; Tronick & Beeghly, 2011).

In atypical development, disruptions to sensorimotor coordination—as in autism spectrum conditions—can interfere with Meaning-Event formation and integration, affecting language, learning, and social understanding (Bruinsma et al., 2004; Sodian & Kristen-Antonow, 2015). The Meaning-Event framework provides a way to conceptualize and analyze these challenges without reducing them to either biological deficits or symbolic impairments alone, emphasizing interactions of biologic and contextual factors of developmental divergence (Diamond & Ling, 2016; Mundy, 2018; Feldman, 2015).

### 6.3. Scope, Limitations, and Future Directions

This article is theoretical and does not present new empirical data (although the initial insights for joint attention as the structure for Meaning-Events (Shaw, 2002, 2018, 2022) are based on Harvard-based research with Kurt Fischer). Its contribution lies in clarifying a conceptual structure for understanding sensorimotor experience, cognition, and meaning as a single developmental process. Future research could operationalize Meaning-Events precisely, develop coding schemes for their sensorimotor components, and examine how Meaning-Views integrate over time (Granott & Parziale, 2002; Mascolo, 2020a).

An obvious extension to this work is how the Meaning-Event structures *joint intention* and *joint action* as well as joint attention. Most references to joint attention in this article could be extended to joint intention and joint action, because the object of attention and action of the meaning-makers, which is built into the Meaning-Event structure, is key to the meaning of the Meaning-Event.

Further work may explore and compare Meaning-Events functioning in multi-person interactions, culturally diverse contexts, narrative identities, and technologically mediated environments. Integrating longitudinal methods, AI modeling, and machine-learning approaches may help elucidate how recursive sensorimotor integration supports learning and adaptation across development (Lind et al., 2025; Da Costa et al., 2020; Gilmore et al., 2020; Newen et al., 2018). Additionally, individual differences in many psychological variables, such as personality, could be analyzed through the lens of significance of Meaning-Events through development, for example, possibly explicating turning points for development of the Big Five (OCEAN) personality traits (Soto & Jackson, 2013).

## 7. Conclusions

In summary, the Meaning-Event framework redefines the structure of ‘event’. It offers a way to understand how early sensorimotor experiences continue to organize cognition and meaning across the lifespan within social contexts. By treating Meaning-Events as sensorimotor–cognitive coordinated units of dyad relationships that are recursively integrated into increasingly complex forms of understanding, the model bridges embodied, cognitive, and psychosocial perspectives. This approach aligns with contemporary developmental science by providing a mechanistic account of how sensory and motor experience shape cognitive development. The model of the Meaning-Event may serve as a nascent step towards integrative research on behavior, connecting positions as disparate as event structure, cognitive neuroscience, lifespan developmental theory, education, and psychotherapy.

Meaning-Events are units through which perception, action, affect, and intention are organized into coherent experiences, tracing how early embodied experiences are preserved, reorganized, and elaborated into complex meaning. A central contribution is treating sensorimotor experience as a consistent developmental mechanism. Research demonstrates that bodily coordination—gaze, posture, timing, affective regulation—plays a foundational role in early development and remains active, though increasingly implicit and symbolically scaffolded, in later forms of learning, reflection, and identity formation (Scaife & Bruner, 1975; Tomasello et al., 2005; Fischer & Bidell, 2006; Barsalou, 2008, 2020; Glenberg, 2015; Overton, 2015).

By foregrounding sensorimotor coordination, the framework connects micro-level interactional processes with macro-level outcomes such as learning, language, and identity (Werner & Kaplan, 1984; Overton, 2015). At the same time, it preserves meaning as a central psychological construct, showing how meaning can be both embodied and reflective, immediate and extended. Meaning-Events provide an integrative structure for understanding how embodied engagement remains central to cognition across the lifespan. By treating sensorimotor coordination as an enduring organizer of experience, the framework invites further empirical investigation using emerging methodological tools to examine how Meaning-Events are enacted, integrated, and transformed across development.

## Figures and Tables

**Table 1:** Integration of Erikson psychosocial stages and Fischer developmental tiers.

Erikson Stages						Fischer Tiers
Stage	Age	Positive	VS	Negative	Resolution	Transition in Developmental Tier
Stage 1	0–1	Trust	VS	Mistrust	Hope	Reflexive to **Sensorimotor**
Stage 2	1–3	Autonomy	VS	Shame	Will	Sensorimotor to **Representational**
Stage 3	3–6	Initiative	VS	Guilt	Purpose	Sensorimotor to **Representational**
Stage 4	6–12	Industry	VS	Inferiority	Confidence	Representational to **Abstract**
Stage 5	12–19	Identity	VS	Confusion	Fidelity	Representational to **Abstract**
Stage 6	20–25	Intimacy	VS	Isolation	Love	Representational to **Abstract**
Stage 7	26–64	Generativity	VS	Stagnation	Care	Abstract to **Principle**
Stage 8	65–	Integration	VS	Despair	Wisdom	Abstract to **Principle**

## Data Availability

No new data were created or analyzed in this study. Data sharing is not applicable to this article.
